# Identification of multidrug-resistant *Neisseria gonorrhoeae* isolates with combined resistance to both ceftriaxone and azithromycin, China, 2017–2018

**DOI:** 10.1080/22221751.2019.1681242

**Published:** 2019-10-29

**Authors:** Qianqin Yuan, Yamei Li, Leshan Xiu, Chi Zhang, Yaoyang Fu, Chuanhao Jiang, Lingli Tang, Junping Peng

**Affiliations:** a Department of Laboratory Medicine, the Second Xiangya Hospital of Central South University, Changsha, People’s Republic of China; b National Health Commission Key Laboratory of Systems Biology of Pathogens, Institute of Pathogen Biology, Chinese Academy of Medical Sciences & Peking Union Medical College, Beijing, People’s Republic of China

**Keywords:** *Neisseria gonorrhoeae*, multidrug-resistant, ceftriaxone, azithromycin, phylogeny

## Abstract

The growing multidrug-resistant *Neisseria gonorrhoeae* is a serious global threat to gonococcal therapy. During 2017–2018, we identified a rare multidrug-resistant (ceftriaxone and azithromycin) strain (GC250) and four strains (GC185, GC195, GC196 and GC249) with both resistance to ceftriaxone and decreased susceptibility to azithromycin. All strains belonged to NG-STAR ST1143, including the mosaic *penA*-60.001, which is closely related to ceftriaxone resistance. The characterization of antimicrobial resistance (AMR) determinants and phylogenetic analysis showed these five strains were closely related to internationally spreading ceftriaxone-resistant *N. gonorrhoeae* FC428, but with higher azithromycin MIC. Findings here demonstrated that this clone not only initiated clonal expansion in China, but acquired azithromycin resistance.

In China, the recommended treatment for uncomplicated gonococcal infection is monotherapy with ceftriaxone [1]. However, with the spread of resistance, dual therapy (ceftriaxone and azithromycin) was recommended as first-line treatments for uncomplicated gonorrhea in many countries worldwide [2]. However, during recent years, the multidrug-resistant (mainly ceftriaxone and azithromycin) *N. gonorrhoeae* isolate has been reported in Ireland [3], Denmark [4], UK [5] and Australia [6]. In China, the prevalence of *N. gonorrhoeae* strain with both decreased susceptibility to ceftriaxone and resistance to azithromycin has increased [1]. Here we describe a rare *N. gonorrhoeae* strain (GC250) with resistance to both ceftriaxone (0.5 mg/L) and azithromycin (2 mg/L), and four strains showed decreased susceptibility to azithromycin while resistance to ceftriaxone. To the best of our knowledge, gonococcal strains with such antimicrobial phenotypes have not been reported in China before. All five strains were isolated in Changsha, China during 2017–2018, and all five strains were assigned NG-STAR type ST1143.

The five isolates were named after GC185, GC195, GC196, GC249 and GC250. Four of five patients (social background information of GC185 patient was missing) are heterosexual males and all four reported that they have had unprotected intercourse with their female partners (case GC250’s partner was a commercial sex worker). Because urethritis symptoms occurred about one week after the sexual activity, all four patients went to the urologic surgery clinic of the Second Xiangya Hospital of Central South University in Changsha, China. The results obtained from culture test show all four patients were positive for *N. gonorrhoeae*.

The minimal inhibitory concentration (MIC, mg/L) profiles for five isolates were provided by using the agar dilution method and all MIC information is summarized in Table 1. The resistance standard is in accordance with the interpretive criteria of the European Committee on Antimicrobial Susceptibility Testing (www.eucast.org). Five strains show resistance to ceftriaxone and the GC250 typically exhibited resistance to azithromycin (2 mg/L), the remaining four show decreased susceptibility to azithromycin.

**Table 1.** Molecular characteristics and antimicrobial susceptibility of ceftriaxone and azithromycin resistant Neisseria gonorrhoeae, China.

Isolate	Year	Country	NG-STAR	*mtrR*	23S rRNA	*porB* (120/121)	*penA* allele	NG-MAST	MLST	MIC(mg/L)		PPNG	*bla* type	Reference
										CRO	AZM			
GC185	2017	China	1143	_△_A	WT	G/G	60	New1^a^	1903	1/R	0.5/DS	Yes	135	This study
GC195	2017	China	1143	_△_A	WT	G/G	60	New1	1903	1/R	0.5/DS	Yes	135	This study
GC196	2017	China	1143	_△_A	WT	G/G	60	New1	1903	1/R	0.5/DS	Yes	135	This study
GC249	2018	China	1143	_△_A	WT	G/G	60	New2	7365	0.5/R	1/DS	Yes	1	This study
GC250	2018	China	1143	_△_A	WT	G/G	60	New2	7365	0.5/R	2/R	Yes	1	This study
BJ16148	2016	China	233	_△_A	WT	G/D	60	3435	1903	0.5/R	0.25/S	NR	NR	[9]
FC428	2015	Japan	233	_△_A	WT	G/D	60	3435	1903	0.5/R	0.25/S	Yes	135	[7]
47707	2017	Canada	233	_△_A	WT	G/D	60	1614	1903	1/R	0.5/DS	Yes	NR	[12]
GK124	2017	Denmark	233	_△_A	WT	G/D	60	1614	1903	0.5R	0.5/DS	NR	NR	[4]
A7846	2017	Australia	233	_△_A	WT	G/D	60	1614	1903	0.5/R	0.25/S	Yes	NR	[13]
IR72	2018	Ireland	1133	_△_A	WT	G/N	60	17842	1903	0.5/R	0.38-0.5/DS	NR	NR	[3]
G97687/ G7944	2018	England	996	_△_A/G45D	A2059G (4 copies)	G/D	60	16848	12039	0.5/R	> 256 /HLR	NR	NR	[5]
A2543	2018	Australia	996	_△_A/G45D	A2059G (4 copies)	G/D	60	16848	12039	0.5	> 256 /HLR	NR	NR	[6]
A2735	2018	Australia	996	_△_A/G45D	A2059G (4 copies)	G/D	60	16848	12039	0.25	> 256 /HLR	NR	NR	[6]

^a^new type, ^b^98% similarity,

R, resistance; S susceptibility; DS, decreased susceptibility; CRO, ceftriaxone; AZM, azithromycin;

WT, Wild type; HLR, High level resistance.

_△_A, a single nucleotide (A) deletion in mtrR promoter.

NR, Not reported.

The sequence types (STs) were identified by using *N. gonorrhoeae* multiantigen sequence typing (NG-MAST), multilocus sequence typing (MLST) and *N. gonorrhoeae* Sequence Typing for Antimicrobial Resistance (NG-STAR) methods (Table 1). The results of NG-MAST contained two new types (*porB*3462, *tbpB*21 and *porB*10477, *tbpB*21) that were not included in the NG-MAST website. MLST results showed that three strains (GC185, GC195 and GC196) belonged to MLST ST1903, which was identical to FC428 [7] and other FC428-like strains. The other two strains (GC249 and GC250) belonged to ST7365, which was a common clone in China [8]. The NG-STAR types of all strains were ST1143 and characterizations of AMR determinants are as follow: mosaic *penA*-60.001 allele, wild type 23S rRNA, a single nucleotide (A) deletion in *mtrR* promoter, G120K -A121G in *por B*, S91F - D95A in *gyrA*, L421P in *ponA*, and S87R in *parC*. Although the NG-STAR STs of the five isolates differed from both FC428 (ST233) and IR72 (ST1133), the difference was only in the A121 position on *porB* gene (Table 1). Moreover, plasmid sequencing was performed on these isolates, showing that all five isolates here were penicillinase-producing *Neisseria gonorrhoeae* (PPNG) strains and GC185, GC195, GC196 contained a TEM-135 β-lactamase gene (Table 1).

In 2016, the FC428 clone (BJ16148) was identified in China, which was resistant to ceftriaxone without azithromycin resistance [9]. Among five strains of this report, three (GC185, GC195 and GC196) shared the same MLST type (ST1903) with the following ceftriaxone-resistant strains, FC428 (Japan), 47707 (Canada), GK124 (Denmark), A7846 (Australia) and IR72 (Ireland). The MLST type of the other two strains (GC249 and GC250) showed high similarity with ST1903, except for only one SNP in one of seven MLST loci (*fumC*). In addition, when there is no difference in other AMR determinants (*mtrR*, *penA*, *gyrA*, *ponA*, *parC*, 23S rRNA), the NG-STAR ST1143 (*porB* A121G) exhibited higher azithromycin MIC than NG-STAR ST233 (*porB* A121D) and NG-STAR ST1133 (*porB* A121N), which indicates the genotype (MLST ST1903/NG-STAR ST233) may have generated variations during spreading. Compared to the confirmed multi-drug resistant strains with NG-STAR type 996 (G97687/G7944, A2543 and A2735), no known resistance mutations were found on 23S rRNA genes of the five strains [10].

To exactly identify the phylogenetic relationship between these strains, a genome-wide phylogeny was constructed. Briefly, sequencing data obtained from Illumina HiSeq X Ten platform (Annoroad, Beijing, China) or Sequence Read Archive (SRA) were aligned to the reference genome of NCCP11945 and a concatenate superset of refined SNPs relative to NCCP11945 was generated to build the maximum-likelihood phylogeny. Detailed method can be found in the previously study [8]. Phylogenetic analysis indicated that the five gonococcal strains in China are closely related to FC428-like strains and were subdivide into two novel subclades, which are linked with other subclades containing strains from Japan, Canada and Australia (Figure 1) [11]. Sequencing data of the five Chinese strains were deposited in Sequence Read Archive (PRJNA560592).

**Figure 1.**
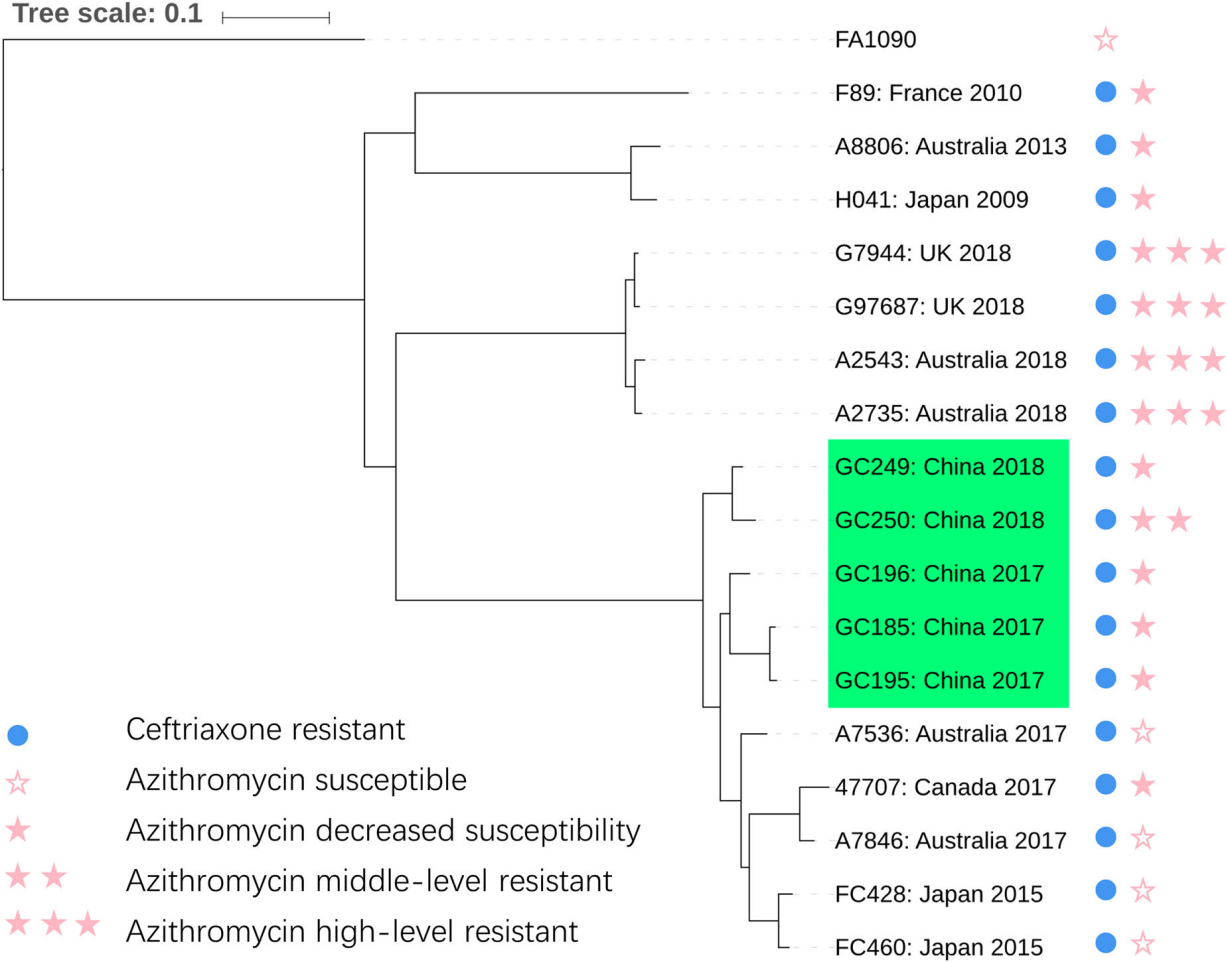
Maximum-likelihood tree based on 14965 genome-wide SNP sites. Strains shaded in green are isolates characterized in this study. Circle and star represent the susceptibility to ceftiaxone and azithromycin respectively. The scale is in the units of mutations per site.

In conclusion, we have identified a rare multidrug-resistant strain with resistance to both ceftriaxone and azithromycin and four strains with decreased susceptibility to azithromycin while resistance to ceftriaxone that are threatening dual-antimicrobial therapy. The results indicated the ceftriaxone-resistant strain FC428 not only initiated clonal expansion in China, but acquired azithromycin resistance.
